# A Simplified Model for Optical Systems with Random Phase Screens

**DOI:** 10.3390/s21175811

**Published:** 2021-08-29

**Authors:** Malchiel Haskel, Adrian Stern

**Affiliations:** Department of Electro-Optics and Photonics Engineering, School of Electrical and Computer Engineering, Ben-Gurion University of the Negev, P.O. Box 653, Beer-Sheva 84105, Israel; stern@bgu.ac.il

**Keywords:** random phase screen, rando phase mask, random medium

## Abstract

A first-order optical system with arbitrary multiple masks placed at arbitrary positions is the basic scheme of various optical systems. Generally, masks in optical systems have a non-shift invariant (SI) effect; thus, the individual effect of each mask on the output cannot be entirely separated. The goal of this paper is to develop a technique where complete separation might be achieved in the common case of random phase screens (RPSs) as masks. RPSs are commonly used to model light propagation through the atmosphere or through biological tissues. We demonstrate the utility of the technique on an optical system with multiple RPSs that model random scattering media.

## 1. Introduction 

An optical system with multiple arbitrary masks placed at arbitrary positions is the core scheme of a variety of optical systems. A scheme where the masks are random phase screens (RPSs) is an excellent model for the analysis, simulation, and interpretation of optical systems that involve optical scattering due to refractive index fluctuations [1,2,3,4]. RPS models were first introduced to represent the fading of radio waves due to fluctuations in the ionosphere layers, but were later found to be useful for modeling various other wave scattering phenomena, including optical scattering. They have been extensively used to analyze atmospheric light propagation, coherent imaging, speckle metrology, and optical scintillations, amongst others [5].

**Figure 1:**
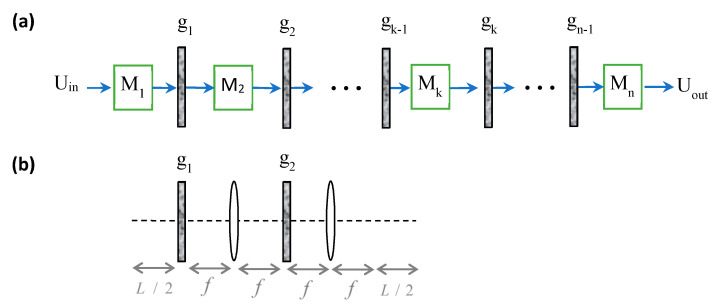
(**a**) A first-order paraxial optical system with n sub paraxial systems $M_{1},M_{2}\ldots M_{k}\ldots M_{n}$ with arbitrary multiple masks $g_{1},g_{2}\ldots g_{k-1},g_{k}\ldots g_{n-1}$ placed at arbitrary positions. $U_{in}$ and $U_{out}$ denote the input field and output field. (**b**) A private case of (**a**) for two random phase masks and three sub-systems. By choosing appropriate system properties, the set-up represents a medium with an optical memory effect.

In Figure 1a, a scheme of a paraxial optical system is illustrated, where there are n sub paraxial systems, $M_{1},M_{2}\ldots M_{k}\ldots M_{n}$, and n−1 masks, $g_{1},g_{2}\ldots g_{k-1},g_{k}\ldots g_{n-1}$, between them, while $U_{in}$ and $U_{out}$ denote the input field and output field, respectively. For example, in atmospheric propagation of light, the sub-systems might be free-space propagation combined with an optical scaling system where the masks are RPSs [4,6]. Figure 1b is a specific case of Figure 1a for two masks and three sub-systems, showing a paraxial system composed of intermittent lenses of focal length f and free-space propagation sections of length L/2 with two masks in between. By choosing the masks as RPSs with properties appropriate for the relevant medium, this figure represents a model [5] for the analysis of the optical memory effect of scattered light in random media [7]. We use Figure 1b as a case study throughout this paper. The basic principle behind the optical memory has its roots in astronomy and was adapted to other fields using well-established astronomic techniques [5,8]. Some other advanced optical memory effect applications might include imaging through the atmosphere, dense fog, or biological tissues [9].

The first order paraxial sub-systems between the masks in Figure 1a might be any combination of beam propagation forms with ideal optical elements between the stages, such as ideal thin lenses, ideal graded-index fiber, etc. Such lenses and fibers are commonly used for scaling [4], imaging [10], and spatial filtering [5] between the RPSs in the system. For example, in Figure 1b, $M_{1}$ is a free-space propagation section of length L/2, $M_{2}$ is an ideal 2f focal length system, and $M_{3}$ is an ideal 2f focal length system with an additional free-space propagation section of length L/2. Thus, we define the optical system without the masks as the “ideal core” optical system. The model may also contain a combination of RSPs with random absorbing screens.

Generally, masks in optical systems have non shift-invariant (SI) behavior; thus, the individual effect of each mask on the output cannot be entirely separated. However, we will show in this paper that the effect of each RPS in the system in Figure 1a can be fully separated under certain conditions. This separation facilitates the analysis of various systems, and helps to understand the relation between the input and the output. Furthermore, it enables separating the influence of each of the system’s parameters and enables modeling more complex systems, such as random media with varying behavior along the system path.

This paper is structured as follows. In Section 2, we investigate the general case of two RSPs, and we demonstrate our approach for the specific case study illustrated in Figure 1b. For completeness of the analysis and for the purpose of comparison, we first review the case of a deterministic mask (Section 2.1), and then we develop the main result of the RPS case and demonstrate it by simulation (Section 2.2). In Section 3, we generalize the approach to the multiple RPS case and its applications. Section 4 presents a discussion and conclusions. 

## 2. Separating the Influence of Individual Masks in an Optical System 

### 2.1. Review of Optical Systems with Deterministic Masks 

**Figure 2:**
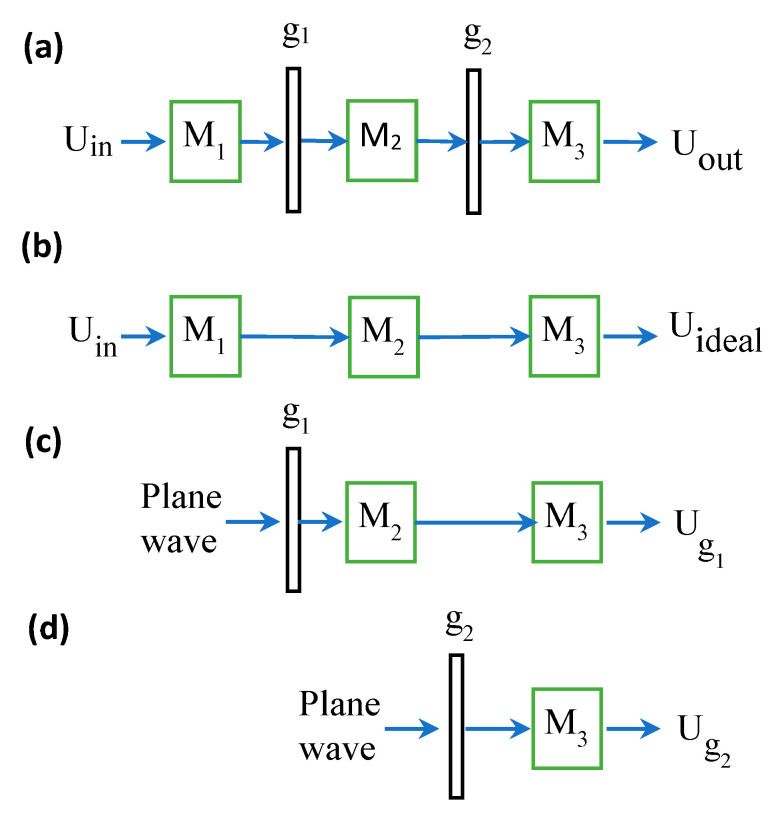
(**a**) General scheme of paraxial optical system with arbitrary general masks (not necessarily RPSs) at arbitrary positions. $M_{1},\;M_{2},\;M_{3}$ denote the sub-systems of the paraxial system before, between, and after the masks $g_{1}$ and $g_{2}$. (**b**) The maskless ideal core system. (**c**,**d**) are the projection systems of $g_{1}$ and $g_{2}$., respectively.

In Figure 2a, a special case of Figure 1a is shown for two masks and three sub-systems; here, the masks are considered to be any general known masks and are not necessarily RPSs. General masks have various optical applications including incoherent holography [11,12], compressive sensing [13], and spectral processing [14]. $M_{1},M_{2}\;$and $M_{3}$. represent sub-paraxial systems before, between, and after masks $g_{1}$ and $g_{2}$, where $U_{in}$ denotes the coherent input and $U_{out}$ denotes the coherent output. In Figure 2b, the scheme of the ideal core system, without the masks, is shown; its coherent output is denoted by $U_{ideal}$. The output field $U_{g_{1}}$ in Figure 2c denotes the projection of the mask $g_{1}$ through the ideal core system from its position to the output plane. Similarly, $U_{g_{2}}$ in Figure 2d is the output projection of the mask $g_{2}$. The output field $U_{out}\;$can be calculated by a sequence of generalized convolutions [15] of the output field core system$\;U_{ideal}$ with the projection output of $U_{g_{1}}$ and $U_{g_{2}}$, which are defined mathematically in Appendix A. Although the generalized convolution provides an elegant and compact way to express the output field, in this paper, we will carry out our analysis using conventional convolutions (the generalized convolution can be expressed in terms of conventional convolutions [16,17]). The output field $U_{out}$ in the scheme in Figure 2a is obtained, up to a multiplication factor, as [18]:

(1) $$U_{out}=q_{3}^{*}\left(\left({\tilde{q}}_{3,32}^{\prime }\left(\left(q_{32}U_{ideal}\right)*V\left[\frac{1}{\lambda b_{32}}\right]{ℑ}^{-1}\left[g_{1}\right]\right)\right)*V\left[\frac{1}{\lambda b_{3}}\right]{ℑ}^{-1}\left[g_{2}\right]\right)$$

where $g_{1}=g_{1}\left(x\right)$, $g_{2}=g_{2}\left(x\right)$, λ is the wavelength, * is the convolution operator and$\;{ℑ}^{-1}$ is the inverse Fourier Transform (FT) operator. $V\left[1/\lambda b\right]h\left(x\right)$ is a scaling operator on a general complex function, with$\;h\left(x\right)$ defined as $V\left[1/\lambda b\right]h\left(x\right)=h\left(\left(1/\lambda b\right)x\right)V\left[1/\lambda b\right]$ [19,20]. For example, a scaled optical FT of mask $g\left(x\right)$ by a 2f system is given, up to a complex factor, by $V\left[-\frac{1}{\lambda f}\right]ℑ\left[g\left(x\right)\right]=\int_{-\infty }^{\infty }g\left(x^{\prime }\right)\exp\left(-j\frac{2\pi }{\lambda f}xx^{\prime }\right)dx^{\prime }$. The quadratic phases in Equation (1) are defined as$\;q_{32}=\exp\left(-\frac{j\pi }{\lambda }\frac{d_{32}}{b_{32}}x^{2}\right),q\prime_{3,32}=\exp\left(-\frac{j\pi }{\lambda }\left(\frac{d_{3}}{b_{3}}-\frac{d_{32}}{b_{32}}\right)x^{2}\right)$ and $q_{3}^{*}$ is the complex conjugate of $q_{3}=\exp\left(-\frac{j\pi }{\lambda }\frac{d_{3}}{b_{3}}x^{2}\right)$, where $b_{3}$ and $d_{3}$ originate from the ABCD ray transfer matrix of the paraxial sub-system $M_{3}$ for the projection of $g_{2}$ onto the output plane (Figure 2d). Similarly, $b_{32}$ and $d_{32}$ originate from the ABCD ray transfer matrix of the cascade sub-system [18] of $M_{2}$ and $M_{3}\;$for the projection of $g_{1}$ onto the output plane (Figure 2c). In Equation (1), and henceforth, we adopt a one-dimensional notation for simplicity, and without loss of generality.

Equation (1) was derived mathematically in [18], but it would be instructive to attribute some physical meaning to it. The term $V\left[\frac{1}{\lambda b}\right]{ℑ}^{-1}\left[g\right]$ plays a similar role to that of the coherent impulse response in a 4f system with g in the Fourier plane [20]. The term q accounts for generalization to other optical systems than the 4f example. In a 4f system, the parameter b is equal to the focal length. For other optical systems, the parameters b and d need to be calculated from the ABCD projection system parameters, as mentioned above. The concept of the forward projections, as shown in Figure 2c,d, can be viewed as a generalization of the projection of clear apertures in geometrical optics. In the geomatical optics approximation, if there are clear apertures in a paraxial system, then it is common practice to project each one towards the image region and the most restricting one is defined as the exit pupil. Then, the scaled FT of the exit pupil can be used to approximate the coherent impulse response. The Fourier scaling parameter is related to the ratio between the aperture and the exit pupil. This conceptual idea is manifested in the field analysis in Equation (1) by the FT of each mask with its own scaling parameter b. The parameter d for each projection is a measure of the shifting of each mask from the Fourier plane, and it approaches zero as the masks are shifted to the Fourier plane.

One advantage of the formalism in Equation (1) is the separation of the influence of $U_{ideal}$ from the expression of $U_{out}$. Unfortunately, only in the particular case of a system with $q_{32}={\tilde{q}}_{3,32}^{\prime }=1$ can the effect of each mask be fully separated from *any other mask* [18], since then Equation (1) reduces to a sequence of pure convolutions and, therefore, the commutative property of the regular convolution operator applies. In this paper, we generalize the effect of each mask and investigate the conditions for such separation for the case of RPSs.

### 2.2. Separating the Effect of Each Random Phase Mask in the Optical System

Consider the case where the masks in Figure 2a are RPSs, and we are interested in the output intensity for a coherent input. The output intensity measurement is the desired result in common optic systems with random media, such as biological and atmospheric light propagation applications [1,2,3,4,21,22]. A practical simulation example of this will be given in the next section. We assume that the RPSs are spatially stationary random processes and that only their statistical properties are known, such as their statistical auto-correlation (AC); therefore, only the average effect of the RPS is measured (Ch.8 in [21]). The commonly used result is the average of many measurements of an ensemble of many RPSs for the same set-up [21]. Consequently, we consider the expected intensity of the system in Figure 2a, modeled by Equation (1) with $g_{1}\;$and $g_{2}\;$being RPSs. The derivation is described in Appendix B, and uses the method for developing the Schell theorem (Chap. 5. in [21]), following well-known techniques for calculating the expected value of a statistical process through linear systems (Ch.7 in [23]). For the derivation, we defined the mask $g\left(x\right)$ as the multiplication of a deterministic mask$\;p\left(x\right)$ with a RPS $t\left(x\right)$, i.e., $g_{1}\left(x\right)=p_{1}\left(x\right)t_{1}\left(x\right)$ and $g_{2}\left(x\right)=p_{2}\left(x\right)t_{2}\left(x\right)$. The deterministic masks $p_{1}$ and $p_{2}$ may represent, for example, simple apertures or diffractive elements. We start with the case of $p_{1}=p_{2}=1$, and we obtain (Appendix B)

(2) $$E\left[\left|U_{out}{}^{2}\right|\right]={\left|U_{ideal}\right|}^{2}*{ℑ}^{-1}\left[\Gamma_{t_{1}}\left(\lambda b_{32}x\right)\right]*{ℑ}^{-1}\left[\Gamma_{t_{2}}\left(\lambda b_{3}x\right)\right]$$

where$\;E\left[•\right]$ is the expected value notation, ${ℑ}^{-1}$. is the inverse FT operator, and $\Gamma_{t_{1}}$ and $\Gamma_{t_{2}}$ are the statistical AC functions of the first and second RPSs $t_{1}$ and $t_{2}$, respectively, where $\Gamma_{t}\left(\Delta x\right)≜E\left[t\left(x\right)t^{*}\left(x-\Delta x\right)\right]$. Thus, the average output intensity $E\left|U_{out}{}^{2}\right|$ is obtained by a regular convolution of the intensity’s ideal core output ${\left|U_{ideal}\right|}^{2}$ with the FT of a scaled AC of each RPS. The parameters $b_{32}$ and $b_{3}$ reflect the influence of the projection of each RPS on the output, according to its position in the system. This influence is analogous to the concept, as described in Figure 2c,d. These are the same scaling parameters of the FT of $g_{1}$ and $g_{2}$ for the deterministic masks in Equation (1). As is evident from Equation (2), owing to the convolution operator’s commutative property, the average effect of each RPS can be entirely separated from any other RPS and the core output intensity ${\left|U_{ideal}\right|}^{2}$. In Equation (2), and henceforth, we ignore constant multiplicative factors. We continue to the case of $p_{1}\neq 1,p_{2}=1$ and we obtain up to complex factor (Appendix B),

(3) $$\begin{array}{cc} \; & E\left[\left|U_{out}{}^{2}\right|\right]=\\ \; & {\left|q_{32}U_{ideal}*{ℑ}^{-1}\left[p_{1}\left(\lambda b_{32}x\right)\right]\right|}^{2}*{ℑ}^{-1}\left[\Gamma_{t_{1}}\left(\lambda b_{32}x\right)\right]*{ℑ}^{-1}\left[\Gamma_{t_{2}}\left(\lambda b_{3}x\right)\right] \end{array}$$

Please note that only the effect of ${ℑ}^{-1}\left[\Gamma_{t_{1}}\left(\lambda b_{32}x\right)\right]*{ℑ}^{-1}\left[\Gamma_{t_{2}}\left(\lambda b_{3}x\right)\right]$ is SI, and not the whole system. Even if the output intensity of the core system ${\left|U_{ideal}\right|}^{2}$ is SI, then, in contradiction to Equation (2), the influence of the deterministic part $p_{1}$ makes the whole core system a non-SI system, i.e., ${\left|q_{32}U_{ideal}*{ℑ}^{-1}\left[p_{1}\left(\lambda b_{32}x\right)\right]\right|}^{2}\neq {\left|q_{32}U_{ideal}\right|}^{2}*{\left|{ℑ}^{-1}\left[p_{1}\left(\lambda b_{32}x\right)\right]\right|}^{2}$. Yet, the effect for each RPS still remains SI (since it is described by a convolution in (3)).

Consider the particular case where the core system is a 4f system, and the input is an on-axis incoherent point source. This is the case in Figure 1b with L=0 and with only one mask, $t_{2}$, located in the Fourier plane, and without $t_{1}$ (i.e., $p_{1}\cdot t_{1}=1$). For this case, Equation (2) reduces to the well-known impulse case of the one random screen analyzing system with an impulse response of (Ch.8 in [21]): ${\left|{ℑ}^{-1}\left[p_{2}\left(\lambda fx\right)\right]\right|}^{2}*{ℑ}^{-1}\left[\Gamma_{t_{2}}\left(\lambda fx\right)\right]$, where ${ℑ}^{-1}\left[\Gamma_{t_{2}}\left(\lambda fx\right)\right]$ is the system average point spread function (APSF), the statistical AC $\Gamma_{t_{2}}\left(\lambda fx\right)$ is the average optical transfer function (AOTF), and the scaled inverse FT of the deterministic AC of $p_{2}$, ${\left|{ℑ}^{-1}\left[p_{2}\left(\lambda fx\right)\right]\right|}^{2}$ is the PSF. Similarly, for the general case in Equation (2), the FT of a scaled AC of each RPS might be considered as its average point spread function (APSF), and their convolution as the total APSF.

We continue to the general case of $p_{1}\neq 1,p_{2}\neq 1$, under the assumption that $p_{2}(x)t_{2}(x)$ is a stationary process. In such a case we may obtain (Appendix B):

(4) $$\begin{array}{cc} \; & E\left[\left|U_{out}{}^{2}\right|\right]=\\ \; & {\left|q_{32}U_{ideal}*{ℑ}^{-1}\left[p_{1}\left(\lambda b_{32}x\right)\right]\right|}^{2}*{ℑ}^{-1}\left[\Gamma_{t_{1}}\left(\lambda b_{32}x\right)\right]*{\left|{ℑ}^{-1}\left[p_{2}\left(\lambda b_{3}x\right)\right]\right|}^{2}*{ℑ}^{-1}\left[\Gamma_{t_{2}}\left(\lambda b_{3}x\right)\right] \end{array}$$

#### Simulation of RPS Separation

**Figure 3:**
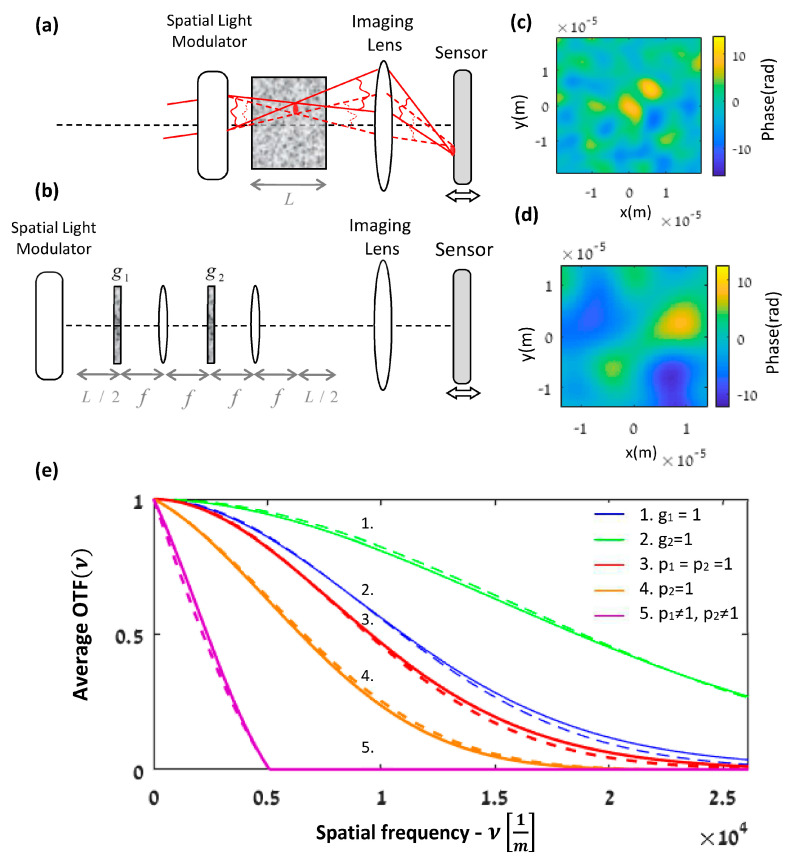
(**a**) Imaging system of scanning and focusing beam inside a medium. (**b**) An equivalent set -up to (**a**) with random phase screens (RPSs), where g=t·p. t is the RPS and p is a deterministic mask. (**c**,**d**) simulations of the first and second RSP, $t_{1}$ and $t_{2}$, with different variance $\sigma_{\phi \prime }^{2}$, for the case study in Figure 1b. (**e**) Average OTF simulation of (**b**) for different values of p and t. (color online), where we choose p to be a clear rectangular aperture. The bold line is the simulation result while the dashed line is the expected analytical result from Equations (5)–(7). (color online).

We shall demonstrate the application of Equations (2)–(4) through the system in Figure 3a. With this scheme, a focusing system focuses a beam inside a random medium and the deep tissue focusing plane is imaged by a lens to a sensor, where the whole imaging system focus is found by moving the sensor. Such a focusing system is commonly applied by using an adaptive optics system [7] based on spatial light modulator for modulating the beam amplitude and phase. Consider the practical case [7] where the focusing system focuses inside a medium that might be represented by our case study in Figure 1b [5]. Thus, Figure 3a can be modeled by Figure 3b. This set-up is a special case of a paraxial system with two RPSs; therefore, Equations (2)–(4) express its APSF.

A simulation analysis of Equations (2)–(4) is clearer in the spatial frequency domain rather than the spatial domain, especially for the following graph visualities purpose. We choose the case where the ideal output is an on-axis point source and by taking the FT of Equations (2)–(4) the AOTF is obtained. For the case of $p_{1}=p_{2}=1$ in Equation (2), it is obtained by

(5) $$AOTF\left(\nu \right)=\Gamma_{t_{1}}\left(\lambda b_{32}\nu \right)\Gamma_{t_{2}}\left(\lambda b_{3}\nu \right)$$

where ν is the spatial frequency variable. The AOTF for the case of $p_{1}\neq 1,p_{2}=1$, in Equation (3) is obtained by

(6) $$AOTF\left(\nu \right)=\left[p_{1}\left(\lambda b_{32}\nu \right)*p_{1}{}^{*}\left(-\lambda b_{32}\nu \right)\right]\Gamma_{t_{1}}\left(\lambda b_{32}\nu \right)\Gamma_{t_{2}}\left(\lambda b_{3}\nu \right)$$

And, similarly, the AOTF for the case of $p_{1}\neq 1,p_{2}\neq 1$ in Equation (4) is obtained by

(7) $$AOTF\left(\nu \right)=\left[p_{1}\left(\lambda b_{32}\nu \right)*p_{1}{}^{*}\left(-\lambda b_{32}\nu \right)\right]\Gamma_{t_{1}}\left(\lambda b_{32}\nu \right)\left[p_{2}\left(\lambda b_{3}\nu \right)*p_{2}{}^{*}\left(-\lambda b_{3}\nu \right)\right]\Gamma_{t_{2}}\left(\lambda b_{3}\nu \right)$$

The medium was modeled according to the case study in Figure 1b, with the RPSs modeled as Gaussian. Following [5], the AC of a Gaussian RPS is $\Gamma_{t}\left(x\right)=\exp\left(-\sigma_{\phi \prime }^{2}x^{2}/2\right)$ where $\sigma_{\phi \prime }^{2}$ is the variance of the derivative of the phase [21]. For our case study, the variances of the first and second RPSs are $\sigma_{1}^{2}=L{\left(2\pi /\lambda \right)}^{2}/l_{tr},\sigma_{2}^{2}=L^{3}{\left(2\pi /\lambda \right)}^{2}/\left(12l_{tr}f^{2}\right)$, respectively [5], where λ is the wavelength, f is the lenses’ focal length in Figure 1b, L is the width of the diffusive medium, and $l_{tr}$ is the transport mean free path. The transport mean free path is associated with the distance through which light propagates between each scattering event and with the anisotropy factor of the medium [3,7]. L/2 is the distance before and after the 4f system in Figure 1b. The system without the masks is just a relay system due to the 4f system. Consequently, the output field is just the result of free-space propagation of the input field by a distance L, as expected for propagation with negligible scattering along the slab. The AOTF accounts for the spatial frequency distribution but does not take into account the intensity loss (by a factor of $\frac{9}{L^{8}}{\left(\frac{\lambda l_{tr}}{\pi }\right)}^{4}$[7]).

Following the experimental conditions reported in [7], we chose L=260 um, $l_{tr}=14.8\;mm$, and λ=632.8 nm. However, it should be emphasized that our case study of the memory effect is valid for any scattering medium or geometry [7]. The focal length f in our model (Figure 1b) is a free parameter that affects the scaling parameter $b_{3}$ in $\Gamma_{t_{2}}\left(x\right)=\exp\left(-{\left(\lambda b_{3}\right)}^{2}\sigma_{2}^{2}x^{2}/2\right)$, but it is balanced by the $\sigma_{2}^{2}$ value. Hence, we choose f to be the same as for all the other cases of free-space propagation in Figure 1b; this choice (i.e., f=L/2) makes it possible to approach the critical sampling for the simulation of free-space propagation [24] and to enhance the simulation accuracy. Consequently, we chose the transfer function simulation approach and not the impulse response approach to better simulate the free-space propagation (Ch.5 in [24]). The Gaussian RPSs were simulated by convolving an uncorrelated random signal with a Gaussian function in a similar way to the Gaussian Schell-model beam simulations [3,24,25].

The Schell theorem is a special case of Figure 1a with one RPS, where p is a clear aperture. Consider p to be a rectangle aperture; then, the diffraction pattern in the Schell theorem is more pronounced as the ratio between the size of p and the transverse coherence length decreases (Chap. 5. in [21]). This is similar to the effect of $p_{1}$ and $p_{2}$ in Equation (4). To demonstrate this principle, we chose a rectangle size of $p_{1}$ and $p_{2}\;$of $2.82\sigma_{1}{}^{-1}$ and $0.31\sigma_{2}{}^{-1}$, respectively.

We simulated the expected value of Equation (1) on the ensemble of 300 different RPSs, but with the same statistical properties. Figure 3c shows one of the RPS simulations for $t_{1}$ from all 300 simulations of the whole ensemble. Similarly, Figure 3d shows one simulation for $t_{2}$ and Figure 3e describes the AOTF simulations after normalization and shows a good match between the simulations and the analytical expressions of Equations (5)–(7). The deterministic OTF of a rectangle aperture has a triangle shape. The fifth graph takes into account the two different size apertures, $p_{1}$ and $p_{2}$, in addition to the Gaussian AOTF of $t_{1}$ and $t_{2}$. This of course reduces the cutoff spatial frequency.

## 3. Generalization for Multiple RPSs

We may generalize the former equations for the system with multiple RPSs shown in Figure 1a. Consider a paraxial system with n sub-systems, $M_{1},M_{2}\ldots M_{k}\ldots M_{n}$, and with n−1 RPSs $t_{1},t_{2}\ldots t_{k-1},t_{k}\ldots t_{n-1}$ between the sub-systems. Then, (2) is generalized to

(8) $$\begin{array}{cc} \; & E\left[\left|U_{out}{}^{2}\right|\right]=\\ \; & {\left|U_{ideal}\right|}^{2}*{ℑ}^{-1}\left[\Gamma_{t_{1}}\left(\lambda b_{n2}x\right)\right]*\ldots *{ℑ}^{-1}\left[\Gamma_{t_{k-1}}\left(\lambda b_{n,k}x\right)\right]*\ldots *{ℑ}^{-1}\left[\Gamma_{t_{n-1}}\left(\lambda b_{n}x\right)\right] \end{array}$$

where $t_{k-1}$ is the k−1 th mask and $b_{n,k}$ originates from the projected ABCD ray transfer matrix of the cascade ideal core sub-systems from the $t_{k-1}\;$position to the output plane.

**Figure 4:**
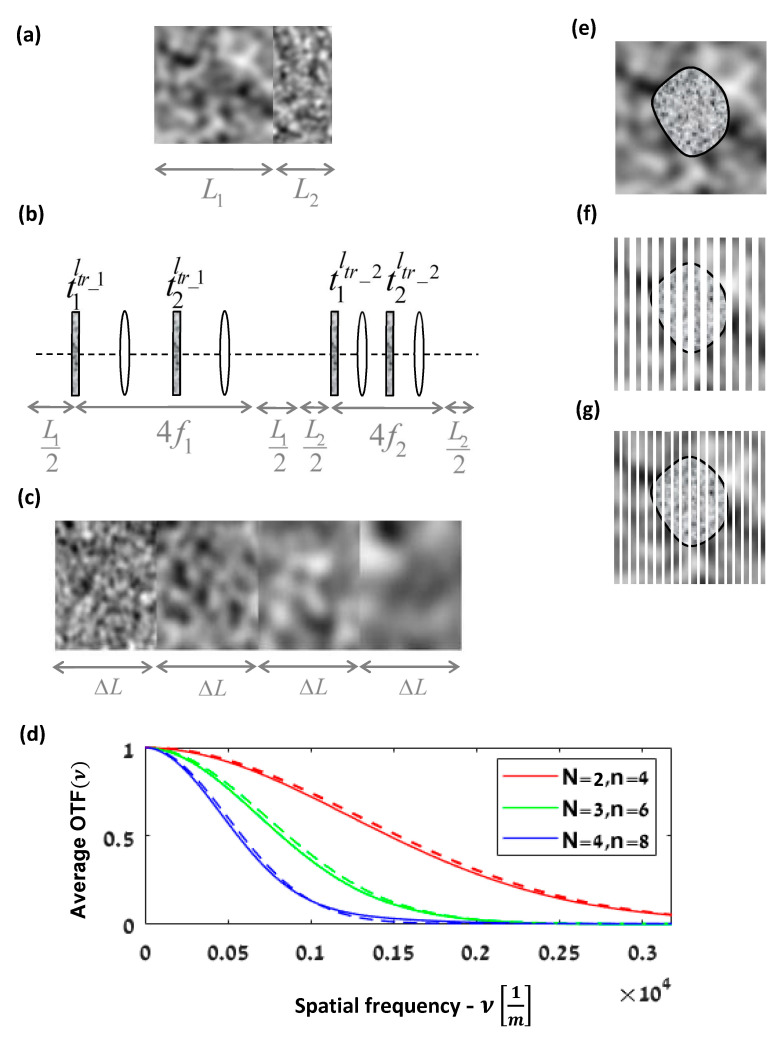
(**a**) Two adjacent media with different lengths and transport mean free path values, $l_{tr}$. (**b**) Equivalent paraxial set-up to (**a**) with RSPs. (**c**) Concatenation of media with gradual change in their $l_{tr}$. (**d**) The AOTF of the particular case where the medium in Figure 3a is replaced by the gradual medium change in Figure 4c. The graphs show the cases of two, three, and four cascaded media. The bold line represents the simulation result while the dashed line shows the expected analytical result from (9). (color online) (**e**) A known mantle random medium in the environment of another random medium. (**f**) Approximation of (**e**) as a stack of many RSPs. (**g**) A higher resolution of (**f**).

This generalization is useful for many cases. For example, Figure 4a can describe the system model of two adjacent random media with different transport mean free paths. Such a combination of random media is modeled by the system shown in Figure 4b, consisting of a cascade of two systems, as seen in Figure 1b, each with appropriate parameters. The specific properties of each medium are represented by different $\sigma_{\phi \prime }^{2}$ values of each Gaussian RPS and its appropriate f and L. In Figure 4b, the RSPs of the first medium are denoted $t_{1}^{l_{tr\_1}}$ and $t_{2}^{l_{tr\_1}}$, with the parameters $f_{1},L_{1}$, and similarly for the second medium; the notations are $t_{1}^{l_{tr\_2}},t_{2}^{l_{tr\_2}},f_{2},L_{2}$.

Although there are now four masks, the overall APSF is still a convolution chain of the effect of each APSF medium, which is one of the advantages of Equation (8). Consequently, for example, if we change the value of $l_{tr}\;$of only one medium, the APSF of only this medium is changed. Similarly, suppose we add a third medium *before* the two media in Figure 4a; in this case, the new overall APSF_overall_ is just a convolution of the overall APSF_two_ of the previous two media with the APSF_third_ of the third medium, i.e., APSF_overall_ = APSF_third_ * APSF_two_. The average effect of each RPS depends on its position in the system in accordance with the scaling factor of the AC. Thus, if we change the order of the two media or their length in Figure 4a, the effect of each RPS is changed only by scaling.

Another useful case is where the random medium properties change along the system path, as is common in vertical atmospheric propagation or in any other scattering scenario that involves changes in the environmental conditions, such as a temperature or pressure gradient. For such a change, the entire system might be seen as cascading of an infinite number of systems of Figure 1b, with gradual changes in their RPS properties. Consider, for example, N adjacent media as described in Figure 4c. Each medium is represented by the case study model in Figure 1b. The RSPs of each medium are Gaussian, with different $l_{tr}\;$according to the medium position. Because each medium is represented by two masks, there are n=2N masks and n+1 sub-systems between them. The value of $l_{tr}\;$for each two masks of the same medium is identical. Considering a gradually linear increase in $l_{tr}$ by a factor of $c_{tr}$, we obtain$\;l_{tr\_K}=Kc_{tr}l_{tr}$, where $l_{tr\_K}$ denotes the $l_{tr}$ of the K_th medium. Each medium has two scaling parameters that depend on their position in the whole system. Assuming all the media have the same length ΔL, and since we may choose the free parameter f to be ΔL/2, as in Section 2.2, we obtain: $b_{n2}=-b_{n3}$, $b_{n3}=\left(n-1\right)\Delta L+\Delta L/2$,$\;b_{n,2K}=-b_{n,2K+1}$,$\;b_{n,2K+1}=\left(N-K\right)\cdot \Delta L+\Delta L/2$,..,$b_{n,2N}=-\Delta L/2$, $b_{n,2N+1}=\Delta L/2$. We denote $\sigma_{1\_K}^{2}$ and $\sigma_{2\_K}^{2}$ as the variance of the first RPS $g_{k}$ and the second RPS $g_{k+1}$ of the same K_th medium, accordingly, where k=K/2 and both variances are a function of the same $l_{tr\_K}$. Thus, using Equation (8), the overall AOTF is obtained by

(9) $$\begin{array}{c} AOTF\left(\nu \right)=\Gamma_{t_{1}}^{l_{tr}{}_{1}}\left(\lambda b_{n2}\nu \right)\cdot \Gamma_{t_{2}}^{l_{tr}{}_{1}}\left(\lambda b_{n3}\nu \right)\ldots \\ \cdot \Gamma_{t_{k}}^{l_{tr}{}_{K}}\left(\lambda b_{n,k}\nu \right)\cdot \Gamma_{t_{k+1}}^{l_{tr}{}_{K}}\left(\lambda b_{n,k+1}\nu \right)\ldots \\ \cdot \Gamma_{t_{N}}^{l_{tr}{}_{N}}\left(\lambda b_{n}\nu \right)\cdot \Gamma_{t_{N+1}}^{l_{tr}{}_{N}}\left(\lambda b_{n+1}\nu \right)\\ =\exp\left(\overset{N}{\underset{K=1}{\sum }}-\frac{{\left(\lambda b_{n,2K}\right)}^{2}}{2}\sigma_{1\_K}^{2}\nu^{2}-\frac{{\left(\lambda b_{n,2K+1}\right)}^{2}}{2}\sigma_{2\_K}^{2}\nu^{2}\right)\\ =\exp\left(\overset{N}{\underset{K=1}{\sum }}-{\left(\lambda b_{n,2K}\right)}^{2}\left(\frac{L_{K}{\left(2\pi /\lambda \right)}^{2}}{l_{tr\_K}}\right)\nu^{2}-{\left(\lambda b_{n,2K+1}\right)}^{2}\frac{{\left(L_{K}\right)}^{3}{\left(2\pi /\lambda \right)}^{2}}{12f^{2}l_{tr\_K}}\nu^{2}\right)\\ =\exp\left(-\frac{4.3\pi^{2}\Delta L}{c_{tr}l_{tr}}\nu^{2}\overset{N}{\underset{K=1}{\sum }}\frac{{\left[\left(N-K\right)\Delta L+\Delta L/2\right]}^{2}}{K}\right) \end{array}$$

Please note that the third equality in Equation (9) is the general AOTF for any N media, each one with its own $l_{tr\_K}$, length $L_{K}$, and projection parameters. Only the last line is the private case for a gradual linear decrease in $l_{tr}$ by a factor $c_{tr}\;$and with the same length $L_{K}=\Delta L$ (Figure 4c). The left summation term in the exponent of the last expression could be considered to be a factor for the average effective $l_{tr}$ of the whole system [26]. Please note that for our case study model (Figure 1b), the dependence of the scattering on the wavelength is assimilated into σ by the $l_{tr}$ value, where $l_{tr}\;$originates from Mie theory [7] and is fundamentally a function of λ. Thus, the dependence of the AOTF on λ is also obtained through $l_{tr}$. Figure 4d shows the AOTF of the particular case where the medium in Figure 3a is replaced by the gradual medium change in Figure 4c. The graphs show the cases of two, three, and four cascading media, i.e., N=2,3,4 and n=4,6,8, where *n* is the number of the masks. For each case of cascaded *N* media, the sensor in Figure 3a was moved to the new imaging plane. We chose each medium length in Figure 4c to be the same as for the value of Figure 3a. The transport mean free path decreases from medium to medium by a factor of $c_{lt}=0.25$. As it can be seen, the graph shows a good match between the simulations and the analytical expression for the multiple masks in Equation (9).

Figure 4a–c are examples for multilayer applications of random media with different properties, which might be analyzed by Equation (8). In the analysis of atmospheric light propagation, this may be relevant when the source or the sensor get closer to or further from each other on the same line of sight. In a biological application, this may be relevant, for example, for evaluating the change in the optical propagation through a multi-layered tissue after removing tissue layers during surgery or due to stretching of the tissue.

In the last examples, we use our case study (Figure 1b) with two RSPs, where the need for multiple RSPs is due to the addition of different media to the system or to a change in the medium properties along the path (Figure 4c) of the same model (Figure 1b). However, multiple RSPs may also be needed for describing other scattering models [1,2,3,6]; thus, the APSF of other multiple RSP models might also be described by Equation (8). For example, it was shown that at least two RPSs are needed to represent a turbulent medium (Ch.9 in [27]). Using only two RSPs enables creating a lab simulator for atmospheric light propagation through two spatial light modulators [4]. However, for the investigation of strong turbulence, a large number of RPSs is needed [6]. Similarly, multiple RSPs are needed to analyze the PSF in the transition regime from ballistic to diffusive light transport [3].

Another valuable application of Equation (8) could be to account for arbitrary shapes of the scattering media. For example, consider the case of a random medium with a known mantle embedded in another random medium (Figure 4e). The multislice method (a.k.a. the beam propagation method) models a three-dimensional object as a series of thin two-dimensional slices separated by homogeneous media. The propagation of the light is analyzed from slice to slice [28]. Therefore, we may approximate the two media as a stack of many RPSs with free-space propagation between them [29]. As the number of slices increases and the distance between them decreases, the approximation is better (Figure 4f). Each slice can be modeled by a combination of three RPSs. One belongs to the inner medium and the others to the environment. If the p of each RPS is known, as well as its statistical AC, then we may use the model. If the scattering of the environment is much smaller than the cell, then we may approximate each slice as a superposition of only two RPSs, one of the environment and the other of the cell.

One of the main technical challenges of the multislice approach is to determine the slice width to be used in the analysis. The common approach is to compute the result with a specific depth resolution, as in Figure 4f, and then, if required, refining by recomputing it with a higher resolution, as depicted in Figure 4g. The refinement process might need to be repeated until convergence to a stable output. In the conventional way, at each refinement step, the output needs to be calculated. However, using our model for computing $\left[\left|U_{out}{}^{2}\right|\right]$, the effect of each slice might be separated from any other slice. Thus, only the additional slices would need to be computed, and their effect is added to the previous computation by the convolution operator. Therefore, the model is effective in the sense of computational efficiency.

## 4. Discussion and Conclusions

The output complex field amplitude of an optical system is formulated in Equation (1) in terms of convolutions of a maskless system with a scaled FT for each mask, with corresponding quadratic phase multiplications. This formulation facilitates separating the effects on the output field of all the masks from the output core system without masks. It also highlights each mask’s influence on the output [18]; however, the effect of each mask on the output cannot be entirely separated from the core system due to the quadratic phase multiplications.

In this paper, we analyze the case where the masks are RPSs. For RPSs, only the statistical properties can be evaluated; thus, we examined the average effect. An elegant and comprehensive approach to analyzing the propagation of the first and second order statistics through the system is by examining the Wigner distribution (WD). For example, in [5], we applied such an approach for the system in Figure 1b. The WD analysis [5] of $U_{out}$ cannot reveal the effect of each individual mask due to the non-SI nature of the system. However, the statistical averaging mechanism of APSF eliminates the quadratic phase terms in Equation (1) and separates the statistical effect of each mask. We have developed analytical expressions, and validated with simulations that this mechanism enables separating the statistical effect of each RPS from that of any other RPS and from the core system.

We conclude by outlining a remarkable difference between systems with deterministic masks and systems with RPS masks. For deterministic masks, the main reason for the SI of $U_{out}$ is the masks’ positions. This affects the quadratic phase factors between the convolutions in Equation (1), and, even if $U_{ideal}$ is generally a SI system, $U_{out}\;$is a non-SI system. In contrast to deterministic mask systems, for the RPS system, the reason for the non-SI feature of the averaged output $E\left[{\left|U_{out}\right|}^{2}\right]$ is the non-SI feature of ${\left|U_{ideal}\right|}^{2}$ and not the RPS positions.

The presented results are useful for exploring various optical systems with multiple-scattering media, and for cases when the random medium properties are not fixed [30] and vary along the system path. The method applies to any paraxial core system and any cascaded system involving different medium properties. For example, the third equality in (9) is valid for any varying function in $l_{tr}$ along the path in Figure 4c, and additional optical systems might be added before, between, and after the media. The method also applies for other models of scattered light in random medium analysis by RSPs, in addition to the specific model in Figure 1b.

There are many different approaches to physically modeling a random medium [1,3,7,22,29]. This paper has made no new contribution to the physical scattering model at the structural level. Rather, the contribution of this paper is at the system model level by separating the RSPs’ effects. We generalize the analysis of any paraxial system with deterministic masks to the case of RPSs. Our model applies to a general framework; it can handle any paraxial core system and any cascaded system of sub-systems with RPSs whose ACs are known. Any paraxial system is defined by the linear canonical transform (LCT) [31,32] and the model is valid for any RPS that is a wide-sense stationary (WSS) process. The generalized LCT convolution and the WSS process have many other application areas beside optics [15,16]. Therefore, the results are valid for any cascaded LCT sub-system with multiplication with WSS processes between the LCT sub-systems.

## Appendix A. The Generalized Convolution

Any paraxial system M can be represented by a corresponding ABCD ray transfer matrix M with the parameters a,b,c,d. Let us denote the sub-systems $M_{1},M_{2},$ and $M_{3}$ in Figure 2 with the corresponding matrices, $M_{1},M_{2},$ and $M_{3}$, respectively. The matrices can be cascaded, so we will denote the maskless system in Figure 2b by the matrix $M_{31}=M_{3}M_{2}M_{1}$ with parameters $a_{31},b_{31},c_{31},d_{31}.$ Similarly, we denote the projection system in Figure 2c by the matrix $M_{32}=M_{3}M_{2}$ with parameters $a_{32},b_{32},c_{32},d_{32}$. The matrix of the projection system in Figure 2d is $M_{3}$, with parameters $a_{3},b_{3},c_{3},d_{3}$.

The ray matrix parameters can be used for the evaluation of the field by virtue of the generalized form of the Kirchhoff–Huygens diffraction integral through a general ABCD system, which is also known as LCT [31,32,33,34,35]. The relation between the output field and the input field of a first order optical system is given, up to complex factor, by

(A1) $$O\left(U_{in}(x)\right)(u)=1/\sqrt{j\lambda b}\int_{-\infty }^{\infty }U_{in}(x)\exp\left[j\frac{\pi }{\lambda b}\left(du^{2}-2ux+ax^{2}\right)\right]dx\;$$

with the parameters a,b,c,d of its corresponding ABCD matrix M. Thus, we denote the same subscript for O and M. $U_{out}$ is obtained by cascading the integral three times with the parameters of the corresponding matrices $M_{1}$,$\;M_{2}$**,** and $M_{3}$, together with multiplication of the masks, i.e., $U_{out}=O_{3}\left(O_{2}\left(O_{1}\left(f_{0}\right)\cdot g_{1}\right)\cdot g_{2}\right)$. The integral has the same cascading property as the matrices. Hence, as with $M_{31}=M_{3}M_{2}M_{1}$ where there are no masks ($g_{1}=g_{2}=1$), we obtain $U_{ideal}=O_{3}\left(O_{2}\left(O_{1}\left(U_{in}\right)\right)\right)=O_{31}\left(U_{in}\right)$ with parameters $a_{31},b_{31},c_{31},d_{31}$ (Figure 2b), and, similarly, $O_{3}\left(O_{2}\left(g_{1}\right)\right)=O_{32}\left(g_{1}\right)=U_{g_{1}}$ (Figure 2c) and $O_{3}\left(g_{2}\right)=U_{g_{2}}$ (Figure 2d).

Similarly to the convolution theorem for FT, a generalized convolution can be defined in the LCT [36], i.e., $O\left(f✶g\right)=O\left(f\right)O\left(g\right)\;$and$\;O\left(f\cdot g\right)=O\left(f\right)✶O\left(g\right)$, where the generalized convolution operator ✶ depends on the O . parameters. By applying this operator three times to calculate $U_{out}$, we obtain:

(A2) $$\begin{array}{cc} U_{out} & =O_{3}\left(O_{2}\left(O_{1}\left(U_{in}\right)\cdot g_{1}\right)\cdot g_{2}\right)=O_{3}\left(O_{2}\left(O_{1}\left(U_{in}\right)\cdot g_{1}\right)\right)✶O_{3}\left(g_{2}\right)\\ \; & =O_{3}\left(O_{2}\left(O_{1}\left(U_{in}\right)\right)✶O_{2}\left(g_{1}\right)\right)✶O_{3}\left(g_{2}\right)=O_{31}\left(U_{in}\right)✶O_{32}\left(g_{1}\right)✶O_{3}\left(g_{2}\right)\\ \; & =U_{ideal}✶U_{g_{1}}✶U_{g_{2}} \end{array}$$

The advantage of the generalized convolution is pronounced in the LCT domain [15,36]. However, $O\left(U\cdot g\right)$ can also be expressed with regular convolution with a quadratic phase before and after the convolution [16,17]; then, $U_{out}$ is obtained by Equation (1) [18].

## Appendix B. Derivation of Equation (4)

Using the convolution theorem, we may rewrite Equation (1) as

(A3) $$U_{out}=q_{3}^{*}{ℑ}^{-1}\left\{h_{2}\left(x\right)g_{2}\left(\lambda b_{3}x\right)\right\}$$

where we denote $h_{2}\left(x\right)=h_{1}\left(x\right)*Q_{32}\left(x\right)$, $Q_{32}\left(x\right)=\exp\left(-\frac{j\pi }{\lambda }{\left(\frac{d_{3}}{b_{3}}-\frac{d_{32}}{b_{32}}\right)}^{-1}x^{2}\right)$, $h_{1}=h_{0}\cdot g_{1}\left(\lambda b_{32}x\right)$ and $h_{0}=ℑ\left[q_{32}U_{ideal}\left(x\right)\right]$. The calculated intensity of Equation (A3) is

(A4) $$\begin{array}{ll} {\left|U_{out}\right|}^{2} & ={\left|q_{3}^{*}\cdot {ℑ}^{-1}\left\{h_{2}\left(x\right)g_{2}\left(\lambda b_{3}x\right)\right\}\right|}^{2}\\ \; & ={ℑ}^{-1}\left\{\left[h_{2}\left(x\right)g_{2}\left(\lambda b_{3}x\right)\right]*{\left[h_{2}\left(-x\right)g_{2}\left(-\lambda b_{3}x\right)\right]}^{*}\right\}\\ \; & ={ℑ}^{-1}\left[\int_{-\infty }^{\infty }h_{2}\left(u\right)g_{2}\left(\lambda b_{3}u\right)h_{2}{}^{*}\left(u-x\right)g_{2}{}^{*}\left(u-\lambda b_{3}x\right)du\right] \end{array}$$

where we use the explicit expression of the convolution integral with a dummy variable u. We assume here that $p_{1}=1$ and we calculate the expected value:

(A5) $$\begin{array}{c} E\left[{\left|U_{out}\right|}^{2}\right]=\\ =E\left[{ℑ}^{-1}\left[\int_{-\infty }^{\infty }h_{2}\left(u\right)g_{2}\left(\lambda b_{3}u\right)h_{2}^{*}\left(u-x\right)g_{2}^{*}\left(u-\lambda b_{3}x\right)du\right]\right]\\ ={ℑ}^{-1}\left[\int_{-\infty }^{\infty }E\left[h_{2}\left(u\right)h_{2}^{*}\left(u-x\right)\right]E\left[g_{2}\left(\lambda b_{3}u\right)g_{2}^{*}\left(u-\lambda b_{3}x\right)\right]du\right]\\ ={ℑ}^{-1}\left[\left(\int_{-\infty }^{\infty }E\left[h_{2}\left(u\right)h_{2}^{*}\left(u-x\right)\right]du\right)E\left[g_{2}\left(\lambda b_{3}u\right)g_{2}^{*}\left(u-\lambda b_{3}x\right)\right]\right]\\ =E\left[{ℑ}^{-1}\left[h_{2}\left(x\right)*h_{2}^{*}\left(-x\right)\right]\right]*{ℑ}^{-1}\left[\Gamma_{t_{2}}\left(\lambda b_{3}x\right)\right] \end{array}$$

where, in the second line of Equation (A5), the expectation operator E can be within the integral, because the FT is a linear transform. Because $t_{1}$ and $t_{2}$ are independent random functions, $h_{2}$ and $g_{2}$ are also independent, and we may calculate the expected value of their involved terms separately (the third line). Assuming $p_{2}=1$, then $g_{2}=t_{2}$, and assuming that the spatial statistics of the RPSs are WSS, then the expected value is independent of the dummy variable and can be factored outside the integral (Ch.8 in [21], [25]), where we use the statistical AC definition (fourth line). Now we need to calculate the left term in Equation (A5). Substituting $h_{2}\left(x\right)=h_{1}\left(x\right)Q_{32}\left(x\right)$ in this term, we obtain, up to complex factor, $E\left[{ℑ}^{-1}\left[h_{2}\left(x\right)h_{2}^{*}\left(-x\right)\right]\right]=E\left[{ℑ}^{-1}\left[h_{1}\left(x\right)h_{1}^{*}\left(-x\right)\right]\right]$, where the effect of $Q_{32}\left(x\right)$ is canceled. We calculate the expected value of the last term in the same manner as in Equation (A5):

(A6) $$\begin{array}{c} E\left[{ℑ}^{-1}\left[h_{1}\left(x\right)*h_{1}^{*}\left(-x\right)\right]\right]=\\ =E\left[{ℑ}^{-1}\left[\int_{-\infty }^{\infty }h_{0}\left(u\right)g_{1}\left(\lambda b_{32}u\right)h_{0}^{*}\left(u-x\right)g_{1}^{*}\left(u-\lambda b_{32}x\right)du\right]\right]\\ ={ℑ}^{-1}\left[\int_{-\infty }^{\infty }h_{o}\left(u\right)h_{0}^{*}\left(u-x\right)p_{1}\left(\lambda b_{32}u\right)p_{1}^{*}\left(u-\lambda b_{32}x\right)E\left[t_{1}\left(\lambda b_{32}u\right)t_{1}^{*}\left(u-\lambda b_{32}x\right)\right]du\right]\\ ={ℑ}^{-1}\left[\left(h_{0}\left(x\right)p_{1}\left(\lambda b_{32}x\right)\right)*\left(h_{0}^{*}\left(-x\right)p_{1}^{*}\left(-\lambda b_{32}x\right)\right)\right]*{ℑ}^{-1}\left[\Gamma_{t_{1}}\left(\lambda b_{32}x\right)\right]\\ ={\left|{ℑ}^{-1}\left[h_{0}\left(x\right)p_{1}\left(\lambda b_{32}x\right)\right]\right|}^{2}*{ℑ}^{-1}\left[\Gamma_{t_{1}}\left(\lambda b_{32}x\right)\right]\\ ={\left|{ℑ}^{-1}\left[ℑ\left[q_{32}U_{ideal}\left(x\right)\right]p_{1}\left(\lambda b_{32}x\right)\right]\right|}^{2}*{ℑ}^{-1}\left[\Gamma_{t_{1}}\left(\lambda b_{32}x\right)\right]\\ ={\left|\left[q_{32}U_{ideal}\left(x\right)\right]*{ℑ}^{-1}\left[p_{1}\left(\lambda b_{32}x\right)\right]\right|}^{2}*{ℑ}^{-1}\left[\Gamma_{t_{1}}\left(\lambda b_{32}x\right)\right] \end{array}$$

where we substitute $g_{1}\left(\lambda b_{32}u\right)=p_{1}\left(\lambda b_{32}u\right)t_{1}\left(\lambda b_{32}u\right)$; this time, the involved term of $\Gamma_{t_{1}}$ is outside the integral, similar to $\Gamma_{t_{2}}$ in Equation (A5) and for the same reasons. We are left with the deterministic terms involving $h_{o}$ and $p_{1}$. Then, we substitute $h_{0}=ℑ\left[q_{32}U_{ideal}\left(x\right)\right]$. By substituting this result in the former equations, we obtain Equation (3).

It is well known that the power spectral density of a random variable is related to its averaged power spectrum (Ch.7 in [23]). Using this theorem and assuming $p_{2}t_{2}\;$is WSS, the expression $E\left[g_{2}\left(\lambda b_{3}u\right)g_{2}^{*}\left(u-\lambda b_{3}x\right)\right]\;$in Equation (A5) is outside the integral and, in the same manner, (Ch.3 in [27]) we obtain (4).
